# Erythema nodosum as a result of estrogen patch therapy for prostate cancer: a case report

**DOI:** 10.1186/s13256-015-0776-4

**Published:** 2015-12-14

**Authors:** Christopher Coyle, Stephen Mangar, Paul Abel, Ruth E. Langley

**Affiliations:** 1 MRC Clinical Trials Unit at UCL, Aviation House, 125 Kingsway, London, WC2B 6NH UK; 2 Imperial College Healthcare NHS Trust, Charing Cross Hospital, Fulham Palace Road, London, W6 8RF UK; 3 Department of Surgery, ‘B’ Block Hammersmith Campus, Imperial College Faculty of Medicine, DuCane Road, London, W12 0NN UK; 4 Department of Urology (3N), Charing Cross Hospital, Imperial College Healthcare NHS Trust, Fulham Palace Road, London, W6 8RF UK

**Keywords:** Erythema nodosum, Estrogen patch, PATCH Trial, Prostate cancer, Transdermal estrogen

## Abstract

**Introduction:**

Erythema nodosum is often associated with a distressing symptomatology, including painful subcutaneous nodules, polyarthropathy, and significant fatigue. Whilst it is a well-documented side-effect of estrogen therapy in females, we describe what we believe to be the first report in the literature of erythema nodosum as a result of estrogen therapy in a male.

**Case presentation:**

A 64-year-old Afro-Caribbean man with locally advanced carcinoma of the prostate agreed to participate in a randomized controlled trial comparing estrogen patches with luteinizing hormone-releasing hormone analogs to achieve androgen deprivation, and was allocated to the group receiving estrogen patches. One month later he presented with tender lesions on his shins and painful swelling of his ankles, wrists, and left shoulder. This was followed by progressive severe fatigue that required hospital admission, where he was diagnosed with erythema nodosum by a rheumatologist. Two months after discontinuing the estrogen patches the erythema nodosum, and associated symptoms, had fully resolved, and to date he remains well with no further recurrence.

**Conclusion:**

Trial results may establish transdermal estrogen as an alternative to luteinizing hormone-releasing hormone analogs in the management of prostate cancer, and has already been established as a therapy for male to female transsexuals. It is essential to record the toxicity profile of transdermal estrogen in men to ensure accurate safety information. This case report highlights a previously undocumented toxicity of estrogen therapy in men, of which oncologists, urologists, and endocrinologists need to be aware. Rheumatologists and dermatologists should add estrogen therapy to their differential diagnosis of men presenting with erythema nodosum.

## Introduction

Erythema nodosum classically presents as raised tender nodules in the skin and subcutaneous tissue. These nodules are most commonly distributed on the lower extremities, particularly the pretibial surfaces, but may occur in other anatomical locations including the thighs and extensor aspects of the forearms. Other associated symptoms may include fever, arthralgia, and generalized weakness [1]. Whilst it is thought to represent a hypersensitivity reaction, determining the etiology of erythema nodosum is notoriously difficult, with the resulting uncertainty causing patients additional distress. The underlying causes include infections, (most commonly Group A streptococcus, but also hepatitis B and C, human immunodeficiency virus, tuberculosis, and mycoplasma pneumonia), disease processes (including inflammatory bowel disease, sarcoidosis, and Behçet’s disease), elevated hormonal states (pregnancy), malignancy (for example, Hodgkin’s lymphoma), and medications (estrogens, sulfonamides, penicillins), and up to 55 % of cases are thought to be idiopathic in origin [2].

Erythema nodosum most commonly occurs in young women. A recent Italian case series (*n* = 124) found that the ratio of females to males was 10:1 and the mean age of onset was 39.5 years old [3]. The propensity towards females is at least partially due to estrogen exposure; in this series, 6.5 % of cases occurred during pregnancy and 5.6 % were attributed to a combination of estrogen-based and progesterone-based medications [3].

The toxicity profile of estrogen is established mainly from its use in women. Toxicities specific to females include breast pain, endometrial hyperplasia, and an increased risk of breast and endometrial cancer [4]. Adverse effects unrelated to gender include venous and arterial thromboembolism, cardiovascular morbidity including cerebrovascular accident, and myocardial infarction [4]. The consequences of estrogen therapy in males may be attributed to either the resulting low testosterone levels (loss of libido, impotence, and hot flushes) or to the direct effects of estrogen (gynecomastia).

Most established indications for estrogen therapy are in women, for example, as a treatment for the symptoms and sequelae of estrogen deficiency in post-menopausal women, and in combination with progesterone as a contraceptive. In males, estrogen therapy is currently used in the treatment of male to female transsexuals [5], and transdermal estrogen is currently being investigated as a potential therapy for men with prostate cancer.

Androgen deprivation therapy resulting in testosterone suppression is a key strategy in the management of prostate cancer. This is mainly achieved through the use of luteinizing hormone-releasing hormone (LHRH) analogs, and occasionally by surgical orchidectomy. Historically, oral estrogen (diethylstilboestrol) has been used for androgen deprivation therapy; however, it is not used routinely because of cardiovascular toxicity attributed to first-pass hepatic metabolism [6]. Transdermal administration of estrogen avoids the entero-hepatic circulation and so is expected to mitigate the risk of cardiovascular toxicity [7].

PATCH (Prostate Adenocarcinoma: TransCutaneous Hormones Trial) is a randomized controlled trial of transcutaneous estrogen patches versus LHRH analogs. An early analysis of toxicity in PATCH (*n* = 138) revealed that men allocated estrogen patches experienced gynecomastia (75 %), impotence (57 %), loss of libido (56 %), and hot flushes (25 %) within the first 6 months of using the patches, though most effects were mild (mainly Common Terminology Criteria for Adverse Events [CTCAE] grade 1 or 2) [8]. In addition to these effects, the consequences of estrogen therapy in male to female transsexuals include redistribution of body fat, decreased muscle mass and strength, softening of skin, and decreased terminal hair growth [5]. To date, there have been no reports in the literature of erythema nodosum developing as a result of estrogen therapy in a man.

## Case presentation

**Fig. 1:**
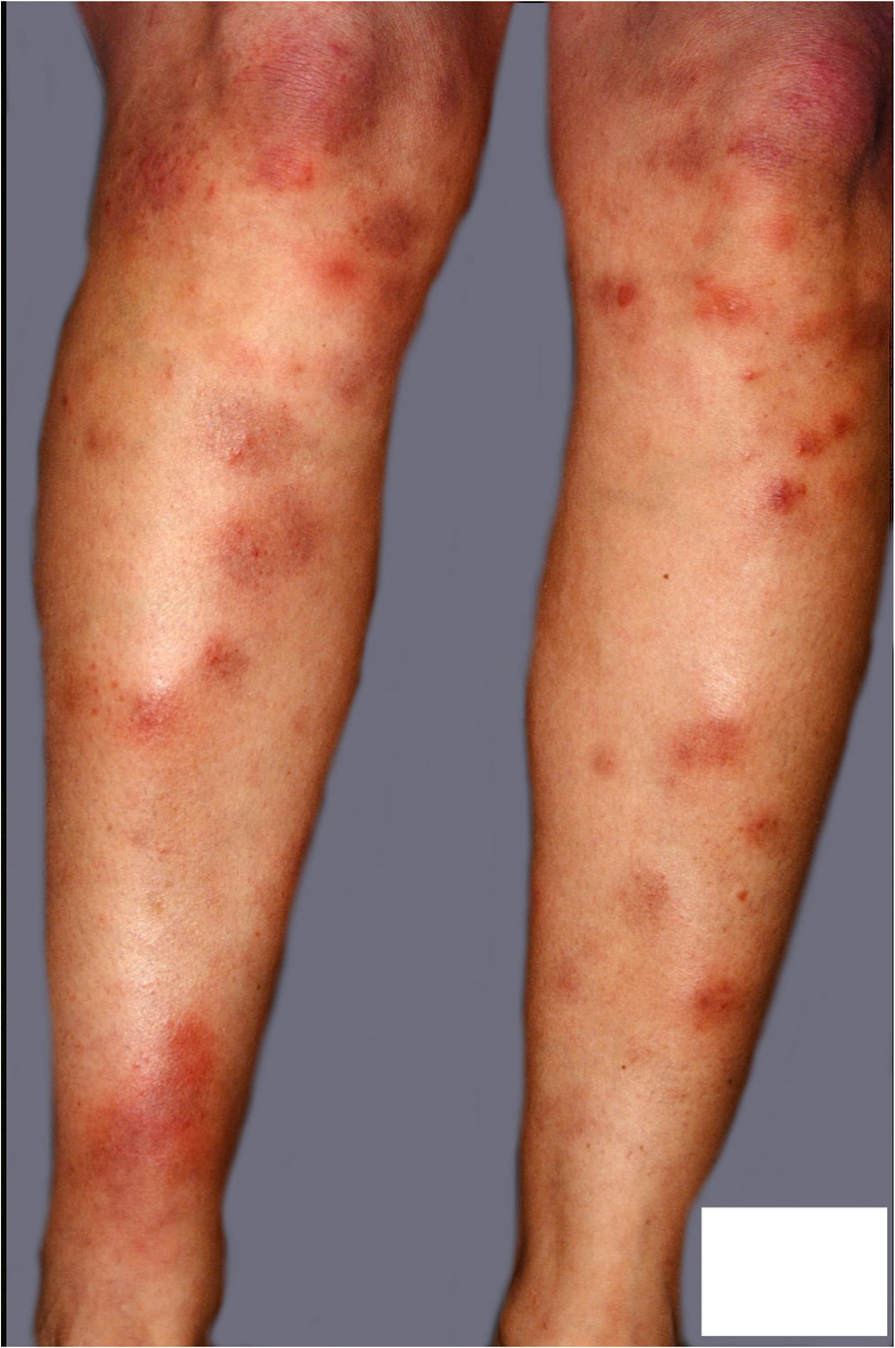
A photograph of bilateral shin lesions taken at the time of first presentation

A 64-year-old Afro-Caribbean man with locally advanced adenocarcinoma of the prostate (T4 NO MO, Gleason score 4+5) was randomized to the transdermal estrogen arm (initially, three Merck Estrogen Femseven patches 100 μg/24 hours, changed twice a week) of the PATCH study (control arm was an LHRH analog). Four weeks later he developed tender lesions on his shins (Fig. 1) associated with painful swelling in his ankles, wrists, and left shoulder, which was followed by progressive severe fatigue. On review by an oncologist, there were initial concerns that the combination of swollen ankles and fatigue could be a result of heart failure secondary to estrogen-induced cardiovascular toxicity. His testosterone level had also fallen to castrate levels, and the number of estrogen patches administered was changed to twice weekly as mandated in the trial protocol. He was then seen by his general practitioner and on examination was found to have bilateral subconjunctival hematomas in addition to the tender nodules on his shins. He was treated with oral diclofenac and misoprostol, oral cephalexin, and chloramphenicol eye drops. His symptoms persisted despite these interventions and 1 week later the decision was made to discontinue the estrogen patches and switch his therapy to an LHRH analog (goserelin).

Two weeks after switching to the LHRH analog, he was admitted to hospital with worsening fatigue and arthralgia. He was found to have sinus tachycardia (115 beats per minute) with no other abnormalities on electrocardiogram, elevated C-reactive protein (122 mg/L, normal <8 mg/L), and elevated alkaline phosphatase (265 u/L, normal range 30–130 u/L). He had recently had a chest X-ray with no abnormal findings, and tested negative for tuberculosis. Streptococcal infection was also excluded.

Our patient was reviewed by a rheumatologist, who confirmed a diagnosis of erythema nodosum as a result of estrogen therapy. His symptoms resolved 8 weeks after discontinuing the estrogen patches (replaced with the LHRH analog), and to date he remains well with no further recurrence. A serious adverse event form was completed describing grade 2 arthralgia and grade 2 peripheral edema (CTCAE v3.0). As a serious and unexpected toxicity of estrogen patches in men, this was reported to the Medicines and Healthcare products Regulatory Agency as a “suspected unexpected serious adverse reaction.”

## Discussion

Attributing the underlying etiology of erythema nodosum relies on the exclusion of other potential causes that fit with the case history. Behçet’s disease may also present with a combination of erythema nodosum and ophthalmic signs, however Behçet’s disease is usually associated with redness and inflammation of the eyes rather than bilateral subconjunctival hematomas, and the absence of any of other features (recurrent mouth and genital ulcers) made this an unlikely diagnosis. Another etiology considered was infection, given the finding of raised C-reactive protein; however, no infective source was identified. Whilst an infectious etiology could not be excluded, given that the initiation and discontinuation of estrogen patches corresponded with the emergence and resolution of erythema nodosum, it seemed highly probable that estrogen was the underlying cause in this case.

## Conclusions

This case report highlights a rare toxicity of estrogen therapy, with only one case recorded, at the time of submission, in 448 patients receiving transdermal estrogen in PATCH with an estimated total of 1120 patient-years of exposure, and no other cases reported in the literature to the best of our knowledge. Transdermal estrogen is already prescribed for male to female transsexuals, and, depending on the findings of PATCH, may be used as an alternative to LHRH analogs in the management of prostate cancer. Transdermal estrogen should be added to the differential diagnosis of men with erythema nodosum, and all health professionals prescribing transdermal estrogen need to be aware of this potential toxicity.

## Consent

Written informed consent was obtained from the patient for publication of this case report and accompanying images. A copy of the written consent is available for review by the Editor-in-Chief of this journal.

## Abbreviations

CTCAE v3.0: Common Terminology Criteria for Adverse Events Version 3.0
LHRH: luteinizing hormone-releasing hormone
PATCH: Prostate Adenocarcinoma: TransCutaneous Hormones trial
